# Supplementary material for: Computational and Experimental Tools to Monitor the Changes in Translation Efficiency of Plant mRNA on a Genome-Wide Scale: Advantages, Limitations, and Solutions
Source: Int J Mol Sci. 2018 Dec 21;20(1):33. doi: 10.3390/ijms20010033 (PMC6337405; doi:10.3390/ijms20010033)
Supplement: Supplementary file 1 [file ijms-20-00033-s001.pdf]

Table S1. Examples of some plant 5'UTRs, their translational activity and advantages, and the used expression systems

5'UTR designation	Origin and plant species	Comparative translation efficiency (assessed according to reporter protein)	Expression system for experimental verification	Advantages	References
**GGR**	*Arabidopsis thaliana*	1.5─2.5-fold higher (in leaves)* 1.3─28-fold higher (in stems)*	Stable expression in *Nicotiana tabacum*	n/d	1
**CA4**	*A. thaliana*	9.7─22.9-fold higher*	Stable expression in *A. thaliana*	Preferential reporter accumulation in mesophyll cells	2
**Gmglnβ1**	*Glycine max*	20-fold higher*	Transient expression in *N. tabacum* var. xanthi	n/d	3
**AtAGP21**	*A. thaliana*	2.5-fold higher (ADH)**	Transient expression in *A. thaliana, Torenia fournieri,* and *N. tabacum* BY2	Translational enhancer for several dicot species	4
**COR47**	*A. thaliana*	1.7-fold higher (ADH)**	Transient expression in *A. thaliana* T87 and *N. benthamiana*	Translational increase under normal and stress conditions	5
**ADH**	*A. thaliana*	n/d	Stable expression in *N. tabacum* and *A. thaliana* T87	Translational increase under normal and stress conditions	6
**Hsp81-3**	*A. thaliana*	5-fold higher (RPS18C)**	Transient expression in *A. thaliana* T87	Translational increase under heat shock conditions	7

* Data of comparison of two reporter constructs with the same promoter fused to the tested 5'UTR and without it.

** Data of comparison of two reporter constructs with the same promoter fused to the tested 5'UTR or to a known translational enhancer (shown in parenthesis).

n/d, not determined.

References

1. Agarwal, P.; Garg, V.; Gautam, T.; Pillai, B.; Kanoria, S.; Burma, P. K. A study on the influence of different promoter and 5′ UTR (URM) cassettes from *Arabidopsis thaliana* on the expression level of the reporter gene β glucuronidase in tobacco and cotton. Transgenic Res. 2014, 23(2), 351-363. doi: 10.1007/s11248-013-9757-9. Epub 2013 Sep 27.

2. Williams, B.P.; Burgess, S.J.; Reyna-Llorens, I.; Knerova, J.; Aubry, S.; Stanley, S.; Hibberd, J.M. An untranslated cis-element regulates the accumulation of multiple 1 C4 enzymes in *Gynandropsis gynandra* mesophyll cells. Plant Cell. 2016, 28(2), 454-465, DOI: https://doi.org/10.1105/tpc.15.00570.

3. Ortega, J.L.; Wilson, O.L.; Sengupta-Gopalan, C. The 5′ untranslated region of the soybean cytosolic glutamine synthetase β 1 gene contains prokaryotic translation initiation signals and acts as a translational enhancer in plants. Molecular genetics and genomics 2012, 287(11-12), 881-893.

4. Matsui, T.; Matsuura, H.; Sawada, K.; Takita, E.; Kinjo, S.; Takenami, S.; Ueda, K.; Nishigaki, N.; Yamasaki, S.; Hata, K.; Yamaguchi, M.; Demura, T.; Kato, K. High level expression of transgenes by use of 5′-untranslated region of the *Arabidopsis thaliana* arabinogalactan-protein 21 gene in dicotyledons. Plant Biotechnol. J. 2012, 29, 319–322.

5. Yamasaki, S.; Matsuura, H.; Demura, T.; Kato, K. Changes in polysome association of mRNA throughout growth and development *in Arabidopsis thaliana*. Plant Cell Physiol. 2015, 56(11), 2169-2180

6. Ueda, K.; Okawara, R.; Yamasaki, S.; Sanada, Y.; Kinoshita, E., Yoneda, A., Demura, T.; Kato, K. Efficient transgene expression by alleviation of translational repression in plant cells. J. Biosci. Bioeng. 2014, 118(4), 434-440.

7. Matsuura, H.; Shinmyo, A.; Kato, K. Preferential translation mediated by Hsp81-3 5′-UTR during heat shock involves ribosome entry at the 5′-end rather than an internal site in Arabidopsis suspension cells. J. Biosci. Bioeng. 2008, 105(1), 39-47.
